# Efficacy and Safety of Shexiang Baoxin Pill for Coronary Heart Disease after Percutaneous Coronary Intervention: A Systematic Review and Meta-analysis

**DOI:** 10.1155/2021/2672516

**Published:** 2021-12-11

**Authors:** Jingjing Wei, Shanshan Liu, Xinlu Wang, Bin Li, Lijie Qiao, Yongxia Wang, Mingjun Zhu

**Affiliations:** ^1^Henan University of Chinese Medicine, Zhengzhou 450000, Henan, China; ^2^The First Affiliated Hospital of Henan University of CM, Zhengzhou 450000, Henan, China

## Abstract

**Objective:**

Shexiang Baoxin Pill (SBP) is a licensed Chinese herbal pharmaceutical that has been widely accustomed to treat coronary heart disease (CHD) after percutaneous coronary intervention (PCI). This study points to systematically assess the efficacy and security of the combination of SBP with conventional western medicine in the treatment of CHD after PCI.

**Methods:**

Databases including PubMed, the Cochrane Library, Web of Science, Embase, CNKI, Wanfang, VIP, and SINOMED were searched to collect RCTs on SBP in CHD after PCI before July 2021. Review Manager 5.3 was used to analyze the data. The Cochrane Collaboration Bias Risk Tool is used to assess the quality of methods.

**Results:**

A total of 19 eligible trials of 2022 patients with CHD after PCI were finally included. The results of the aggregate evidence showed that, compared with routine western medicine treatment alone, the combination of SBP with conventional treatment trial groups could significantly reduce the incidence of major adverse cardiac events (MACE) of the patients (RR = 0.38, 95% CI (0.29, 0.51), *P* < 0.00001). SBP also significantly enhanced left ventricular ejection fraction (LVEF) (MD = 4.00, 95% CI (3.42, 4.58), *P* < 0.00001) and lessened N-terminal pro-B-type natriuretic peptide (NT-pro-BNP) levels (MD = −167.18, 95% CI (−174.80, −159.57), *P* < 0.00001). In addition, the inflammatory mediators and blood lipid index in the experimental group after the combined therapy were also mediated (*P* < 0.05). Moreover, SBP did not increase the incidence of adverse reactions during treatment. The results of subgroup analysis illustrated that the length of the intervention course might be the source of the heterogeneity of NT-pro-BNP and hs-CRP.

**Conclusion:**

SBP could demonstrate a beneficial role in patients with CHD after PCI of reducing the incidence of MACE and improving LVEF, NT-pro-BNP, inflammatory mediators, and blood lipid index. However, limited by the quantity and quality of eligible studies, the above conclusions required more standardized, rigorous, high-quality clinical trials to verify further.

## 1. Introduction

Cardiovascular disease (CVD) is one of the leading causes of morbidity and death worldwide, accounting for approximately 31.5% of global deaths and 45% of noncommunicable disease deaths. Among them, coronary heart disease (CHD) is the primary clinical manifestation of CVD [1, 2]. Percutaneous coronary intervention (PCI) can quickly recanalize the coronary arteries briefly, realize the prevention of vasodilation and secondary stenosis, and effectively improve the patient's myocardial blood perfusion. It has become one of the main methods of clinical treatment for CHD [3].

In 2015, the number of interventional therapies for CHD in Mainland China reached 567,583, ranking second to the United States in the world. It should be noted that, according to the China Cardiovascular Intervention Forum (CCIF) report, this number reached 968,651 in 2020 [4]. However, PCI cannot eliminate the risk factors of CHD, nor can it reverse the progression of coronary atherosclerosis. Stent placement may damage the vascular endothelium, trigger inflammatory reactions, and promote platelet adhesion and aggregation and thrombosis, which may lead to the occurrence of MACE such as in-stent restenosis, recurrence of angina pectoris, arrhythmia, recurrent myocardial infarction, heart failure, and even cardiogenic death, limits the therapeutic effect of PCI, and brings a negative impact on the patient's prognosis. Therefore, there is an urgent need for new methods to decrease the residual risk after PCI and serve as the ultimate alternative for patients intolerant to standard drugs.

Traditional Chinese medicine (TCM) focuses on the overall concept and treatment based on syndrome differentiation, with few toxic and side effects, potentially additional therapy [5]. Shexiang Baoxin Pill (SBP) has been used to treat CHD for nearly 40 years in China. SBP belongs to the national secret Chinese medicine variety, composed of artificial musk, arenobufagin, borneol, storax, cinnamon, ginsenosides, and bufadienolides [6]. Modern pharmacological studies have shown that SBP and its active ingredients have pleiotropic effects in protecting the circulatory system, such as promoting therapeutic angiogenesis, restoring myocardial function, reducing inflammation, and improving endothelial dysfunction, which may be necessary for the curative effect of CHD after PCI. At the same time, evidence-based clinical studies have shown that SBP is advantageous as an adjuvant treatment in numerous cardiovascular diseases, such as stable angina, myocardial infarction, and heart failure [7–9]. However, the clinical evidence regarding the efficacy of SBP in the treatment of CHD after PCI has not been well summarized yet. Therefore, this study adopted systematic review methods to assess the clinical efficacy after the SBP treatment of CHD after PCI to provide sufficient evidence in the clinical decision-making process.

## 2. Materials and Methods

In order to increase the transparency and quality of systematic evaluation reports, this research complied with PRISMA 2020 statement and has been registered in PROSPERO (Registration Number: CRD42021283638).

### 2.1. Inclusion and Exclusion Criteria

#### 2.1.1. Types of Research

RCTs of SBP for patients with CHD after PCI were strictly included and were not restricted by the publishing language. Specific and accurate data can be obtained for analysis.

#### 2.1.2. Object of Study

All subjects meet the relevant diagnostic criteria for CHD established by the American Heart Association or the Chinese Medical Association [10, 11] and successfully accept PCI regardless of age, gender, race, and region.

#### 2.1.3. Intervention Measures

The intervention control group was addressed in the same conventional western medicine only. Conventional western medicines include antiplatelet aggregation drugs, *β*-receptor blockers, anticoagulation, angiotensin-converting enzyme inhibitors, statins, nitrates, and other drugs recommended by the guidelines. At the same time, the treatment group was given SBP combined with conventional western medicine.

#### 2.1.4. Exclusion Criteria

The research that is not rigorous, has incomplete data, or has a significant error, and the statistical analysis that cannot be performed was excluded. The study on the treatment group or the control group with other Chinese herbal medicines, the animal experiments, experience reports, conference papers, and the repeated published pieces of literature that could not get the full text were excluded.

#### 2.1.5. Observation Index

The primary outcome indicators are as follows: ① MACE including recurrent angina, myocardial infarction, malignant arrhythmia, cardiac failure event, and cardiac death; ② left ventricular ejection fraction (LVEF); and ③ N-terminal pro-B-type natriuretic peptide (NT-pro-BNP). The secondary outcome indicators are as follows: ① inflammatory mediators including interleukin- 6 (IL-6) and hypersensitive-C-reactive protein(hs-CRP); ② blood lipid index including total cholesterol (TC), triglyceride (TG), low-density lipoprotein cholesterol (LDL-C), and high-density lipoprotein cholesterol (HDL-C); and ③ adverse reactions.

### 2.2. Retrieval Strategy

Randomized controlled trials of SBP in the treatment of CHD after PCI were searched by the computer system from PubMed, Embase, Web of Science, Cochrane Library, VIP, CNKI, Wanfang, and China Biomedical Database since the establishment of the database until July 2021. In order to ensure comprehensiveness and integrity, the combination of subject words and free words is used to search the literature. Following search terms were used in combination: (“Shexiang Baoxin pill” OR “Heart pill of musk”) AND (“coronary heart disease” OR “CHD” OR “coronary artery disease”) AND (“percutaneous coronary intervention” OR “PCI”).

### 2.3. Literature Screening and Data Extraction

Two investigators (Xinlu Wang and Bin Li) independently read the literature title and screen abstract, rescreened the complete text, extracted essential information for final inclusion, and cross-checked it. In case of disagreement, it will be approved by a third researcher. The extracted contents include the type of study design, baseline characteristics, intervention methods (intervention measures and treatment duration), leading outcome indicators, and adverse reactions [12].

### 2.4. Methodological Quality Evaluation

Two independent researchers (Jingjing Wei and Lijie Qiao) assessed the quality of the included literature according to the risk of bias assessment tool recommended in the Cochrane System Reviewer Manual 5.1.0 [13]. The evaluation content includes randomization process, allocation concealment, blinding of participants and researchers, blinding of outcome assessment, incomplete or missing outcome data, selective reporting, and another risk of bias source. Quality assessment of included studies was separated into “low risk,” “unclear risk,” and “high risk” for the above seven evaluation items. Disagreements were resolved through discussion and decision by Prof. Mingjun Zhu.

### 2.5. Statistical Treatments

Rev Man5.3 software was used only for the statistical analysis of the included literature research data. For continuous variables, if the measurement method is the same as the measuring unit, the mean difference (MD) should be adopted as the effect measure; if the measurement method is different from the measuring unit, the standard mean difference (SMD) should be adopted as the effective measures. Binary variables should use the risk ratio (RR) as a practical measure. Both situations give a 95% confidence interval (CI). If significant heterogeneity was found in the experimental results (*I*^2^*>*50%, *P* ≤ 0.1), the random effect model was used only for meta-analysis; if the experimental results showed good homogeneity (*I*^2^ ≤ 50%, *P* > 0.1), the fixed-effect model was used for meta-analysis. Sensitivity analysis or subgroup analysis was used to explore sources of heterogeneity. The funnel plot was used to determine whether there is a bias risk in the literature.

## 3. Results

### 3.1. Literature Retrieval

**Figure 1:**
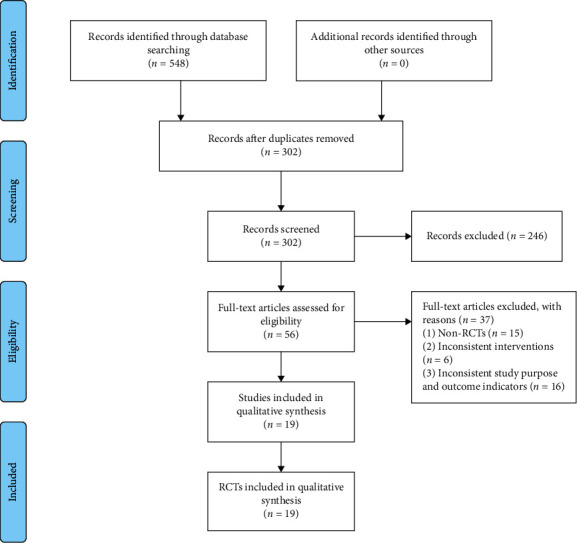
Flowchart of literature retrieval and screening.

Five hundred forty-eight references were initially retrieved from the medical database, including 352 Chinese articles and 196 English articles. After deleting the repeated literature, the remaining 302 articles were excluded after reading the title, abstract, and full text. Finally, 19 RCTs [14–32] were included. The literature screening flow chart and results are shown in Figure 1.

### 3.2. Basic Features of Literature Research

Nineteen eligible studies [14–32] involving 2022 patients were all published in Chinese databases. The number of cases in the experimental group was 1019 and that in the control group was 1003. All studies had clear inclusion and exclusion criteria and reported that the baseline of the experimental and control groups was comparable. The intervention measures of the treatment group in all studies were conventional western medicine combined with the oral administration of SBP, and the intervention measures of the control group were conventional western medicine. All the intervention measures in the treatment group combine SBP and conventional western medicine; simultaneously, the control group's intervention measures are the conventional western medicine therapy. In the observation of outcome indicators, 14 studies [14, 16, 18, 20, 23, 27–32] observed MACE, 9 studies [21, 23, 25, 28–32] observed LVEF, 4 studies [19, 23, 26, 28] observed NT-pro-BNP, 6 studies [15, 18, 22, 25] observed inflammatory mediators, 4 studies [16, 18, 26] observed blood lipid index, and 4 studies [16, 23, 28, 29] observed adverse reactions. The basic features of the 19 eligible literature are shown in Table 1.

### 3.3. Literature Quality Assessment

**Figure 2:**
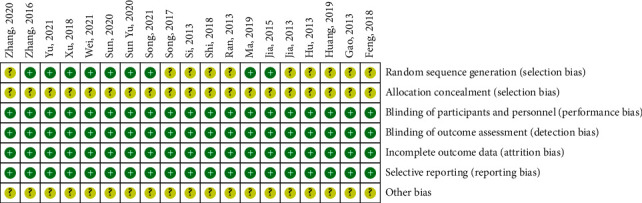
Summary of bias risk assessment for included trials.

All eligible studies are randomized controlled trials, nine studies [14, 16, 23, 25, 27, 28, 31, 32] of which describe the method of randomization process in detail, with random number table as the specific methods, and the risk of bias on the domain was judged as “low risk.” The remaining ten studies [15, 17, 22, 26, 29, 30] reported “random” but did not describe specifically and were assessed as “unclear risk.” All studies that did not mention allocation concealment were assessed as “unclear risk.” Due to the objectivity of outcome indicators, the implementation of the blind method should be considered “low risk,” regardless of whether the blinding was reported. There was no missing outcome data or selective reporting bias in all literature, rated as “low risk.” The results of other biases are unclear, and the specific bias risk assessment information is shown in Figure 2.

### 3.4. Synthesis of Outcome

#### 3.4.1. Major Adverse Cardiac Events

**Figure 3:**
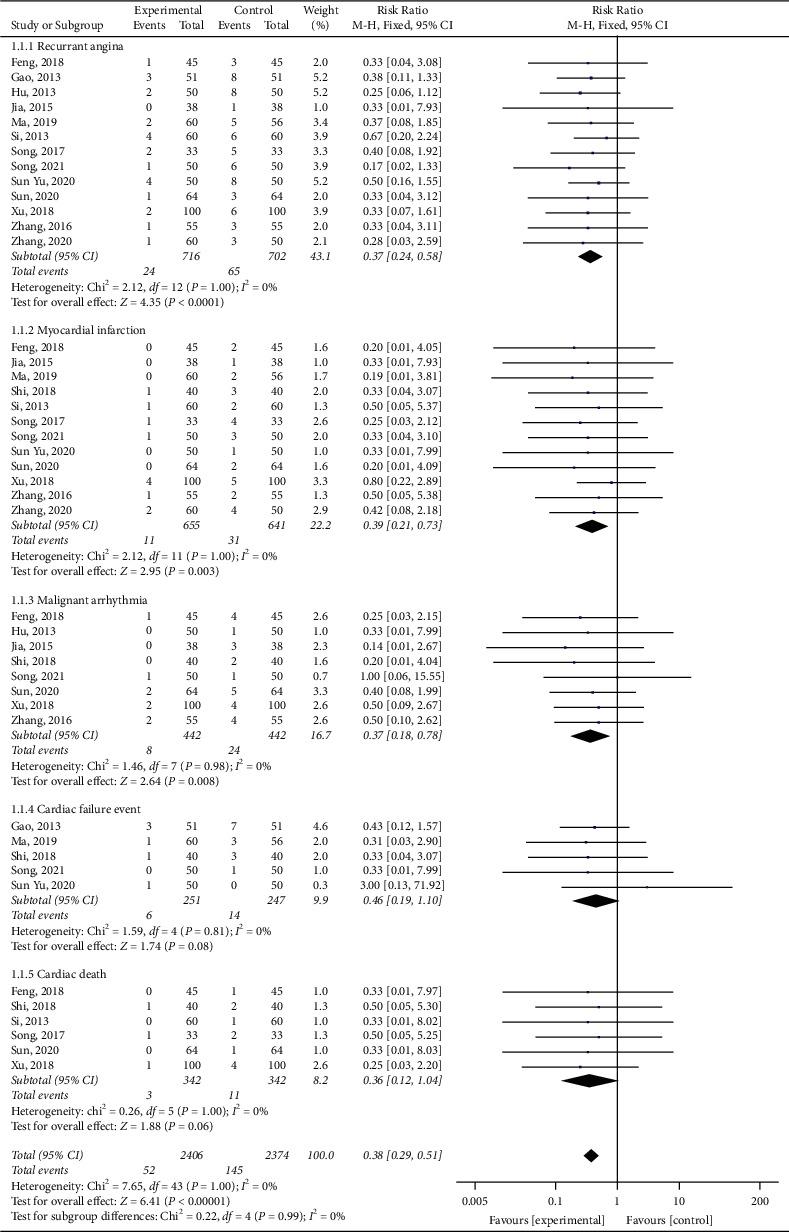
Subgroup analysis of major adverse cardiac events according to different cardiac events.

Fourteen studies out of the 19 studies [14, 16, 18, 20, 23, 27–32] compared the incidence of MACE between the combined therapy and the conventional western medicine therapy alone. No statistically significant heterogeneity (*P*=1.00, *I*^2^ = 0%) was found among 1498 participants, so the fixed effects model meta-analysis results showed a statistically significant difference (RR = 0.38, 95% CI (0.29, 0.51), *P* < 0.00001), suggesting that SBP combined with conventional western medicine reduced the incidence of MACE, which is better than the control group for patients with CHD after PCI (Figure 3). Subgroup analysis was implemented based on different cardiac events. There were recurrent angina, myocardial infarction, malignant arrhythmia, cardiac failure event, and cardiac death. Thirteen studies [14, 16, 18, 20, 23, 27, 29, 31, 32] reported the incidence of recurrent angina, and an appropriate effect model was used for analysis (*P*=1.00, *I*^2^ = 0%). The results showed that the incidence of recurrent angina in the experimental group was significantly lower than that in the control group (RR = 0.37, 95% CI (0.24, 0.58), *P* < 0.0001). Twelve studies [14, 16, 18, 22, 23, 27–32] reported the incidence of myocardial infarction, and an appropriate effect model was used for analysis (*P*=1.00, *I*^2^ = 0%). The results showed that the incidence of myocardial infarction in the experimental group was significantly lower than that in the control group (RR = 0.39, 95% CI (0.21, 0.73), *P*=0.003). Eight studies [14, 16, 17, 20, 23, 27, 28, 30] reported the incidence of myocardial arrhythmia, and an appropriate effect model was used for analysis (*P*=0.98, *I*^2^ = 0%). The meta-analysis showed that the incidence of malignant arrhythmia in the experimental group was significantly lower than that in the control group (RR = 0.37, 95% CI (0.18, 0.78), *P*=0.008). Five studies [14, 16, 18, 20, 23, 27, 29, 31, 32] reported the incidence of the cardiac failure event, and an appropriate effect model was used for analysis (*P*=0.81, *I*^2^ = 0%). The results showed no evidence that the experimental group is better than the control group (RR = 0.46, 95% CI (0.19, 1.10), *P*=0.08). Six studies [14, 16, 18, 29, 30] reported the incidence of cardiac death, and an appropriate effect model was used for analysis (*P*=1.00, *I*^2^ = 0%). The results showed that no statistical difference exists in the incidence of cardiac death in the two groups (RR = 0.36, 95% CI (0.12, 1.04), *P*=0.06) (Figure 3).

**Figure 4:**
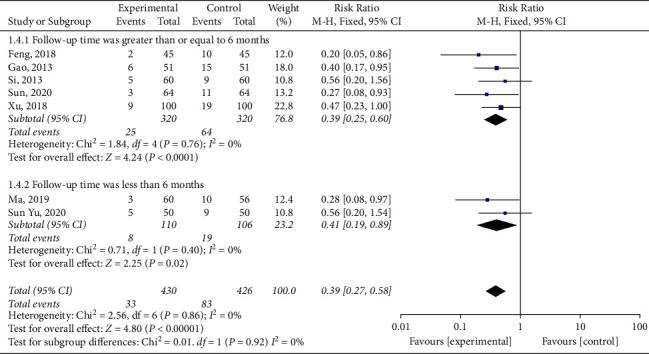
Subgroup analysis of major adverse cardiac events according to the follow-up time.

Furthermore, subgroup analysis was conducted according to the follow-up time, and the heterogeneity of follow-up time greater than or equal to 6 months was small (*P*=0.76, *I*^2^ = 0%) [14, 16, 18, 21]. A fixed-effect model was used for meta-analysis, and compared with the control group, the incidence of MACE in the experimental group was significantly decreased (RR = 0.39, 95% CI (0.25, 0.60), *P* < 0.0001). The heterogeneity of patients with follow-up time less than six months was small (*P*=0.40, *I*^2^ = 0%) [31, 32]. Meta-analysis using the fixed-effect model showed statistically significant difference (RR = 0.41, 95% CI (0.19, 0.89), *P*=0.02), indicating that the experimental group could significantly reduce the incidence of MACE, as shown in Figure 4.

#### 3.4.2. LVEF

**Figure 5:**
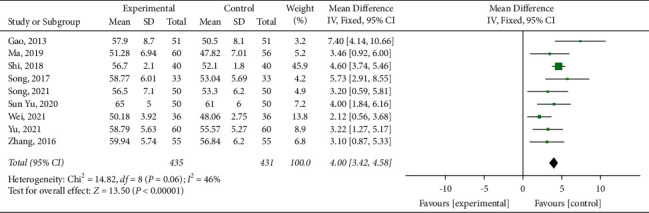
Meta-analysis of the left ventricular ejection fraction.

Nine [21, 23, 25, 28–32] studies reported LVEF, including 435 sufferers in the experimental group and 431 in the control group. A fixed-effect model was adopted to carry out the meta-analysis (*P*=0.06, *I*^2^ = 46%). As a result, as shown in Figure 5, the experimental group had a better effect on improving LVEF than the control group (MD = 4.00, 95% CI (3.42, 4.58), *P* < 0.00001).

#### 3.4.3. NT-pro-BNP

**Figure 6:**
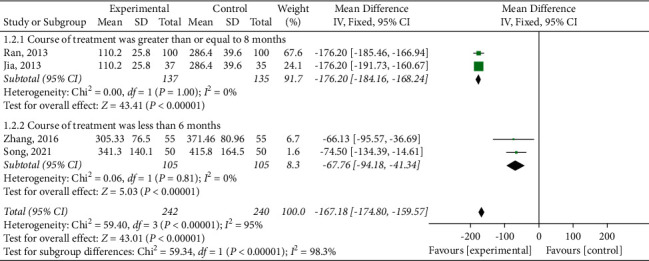
Meta-analysis of N-terminal pro-B-type natriuretic peptide.

Four RCTs reported the assessment results of NT-pro-BNP [ 23, 27, 30, 32]. The intervention course of the drug was crucial to the clinical efficacy. Subgroup analysis was performed according to different intervention courses, as shown in Figure 6. The heterogeneity of subgroups with treatment courses, which are more than or equal to 6 months, was significantly reduced (*P*=1.00, *I*^2^ = 0%) [19, 26], so the fixed effects model meta-analysis results showed that the experimental group could significantly reduce NT-pro-BNP (MD = −176.20, 95% CI (−184.16, −168.24), *P* < 0.00001). When the intervention course was less than six months [23, 28], high homogeneity existed among individual studies (*P*=0.81, *I*^2^ = 0%). An appropriate effect model was used for analysis, and the results showed that the experimental group could significantly reduce NT-pro-BNP (MD = −67.76, 95% CI (−94.18, −41.34), *P* < 0.00001). The results of the subgroup analysis suggested that the length of the intervention course may be the source of heterogeneity of NT-pro-BNP.

#### 3.4.4. Inflammatory Mediators

**Figure 7:**
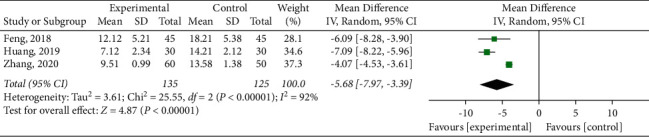
Meta-analysis of interleukin- 6.

Interleukin-6 (IL-6) was observed in three studies [15, 17, 22], and significant heterogeneity was found among the studies (*P* < 0.00001, *I*^2^ = 92%). The results showed that the SBP experimental group was superior to the control group in reducing IL-6 (MD = −5.68, 95% CI (−7.97, −3.39) *P* < 0.00001) (Figure 7). In order to clarify the source of heterogeneity, sensitivity analysis was adopted to exclude literature one by one. When the study of Zhang (2020) was excluded (*P*=0.43, *I*^2^ = 0%), the study was considered the source of heterogeneity. It was found that the more significant heterogeneity might be due to different lengths of the intervention course or publication bias.

**Figure 8:**
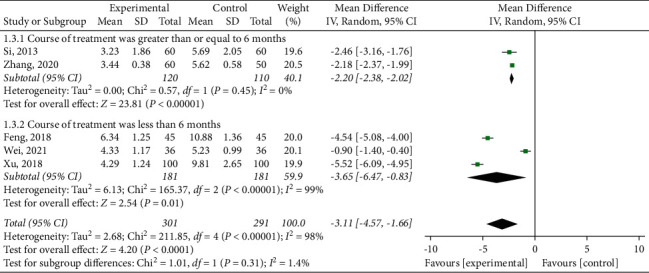
Meta-analysis of hypersensitive-C-reactive protein.

Five RCTs reported the results of hs-CRP evaluation [16, 18, 22, 25], based on the heterogeneity test results (*P* < 0.00001, *I*^2^ = 98%), continued to take different intervention courses for subgroup analysis, as shown in Figure 8. The heterogeneity of subgroups with a course of more than or equal to 6 months was significantly reduced (*P*=0.45, *I*^2^ = 0%) [18,22], so the fixed effects model meta-analysis, the results showed statistically significant difference (MD = −2.20, 95% CI (−2.38, −2.02), *P* < 0.00001), suggesting that SBP combined conventional western medicine to reduce CRP in patients with superior to the control group. When the intervention course was less than six months [16, 17, 25], there was significant heterogeneity between individual studies (*P* < 0.00001, *I*^2^ = 99%). An appropriate effect model was used for analysis, and the results showed that the experimental group could significantly reduce hs-CRP (MD = −3.65, 95% CI (−6.47, −0.83), *P*=0.01). The results of subgroup analysis suggested that the subgroup with the combined intervention course of more than or equal to 6 months had fair homogeneity.

#### 3.4.5. Blood Lipid Index

**Figure 9:**
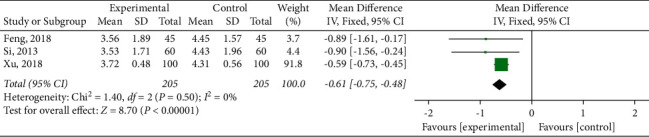
Meta-analysis of total cholesterol.

**Figure 10:**
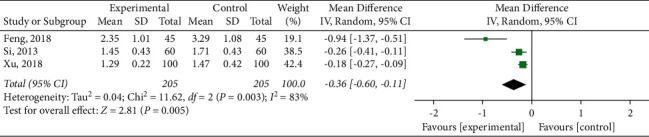
Meta-analysis of triglyceride.

**Figure 11:**
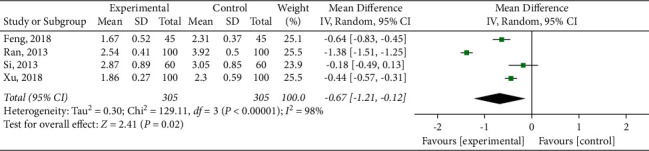
Meta-analysis of low-density lipoprotein cholesterol.

**Figure 12:**
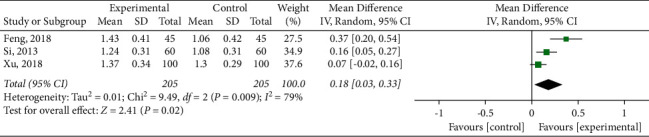
Meta-analysis of high-density lipoprotein cholesterol.

Three studies of all reported the assessment results of TC [16–18], and the studies were homogeneous (*P*=0.50, *I*^2^ = 0%). The meta-analysis results showed that the experimental group could significantly reduce TC (MD = −0.61, 95% CI (−0.75, −0.48), *P* < 0.00001), as shown in Figure 9. Three studies reported the assessment results of TG [16–18]. There was significant heterogeneity among individual studies (*P*=0.003, *I*^2^ = 83%). The results showed that the TG in the experimental group could be significantly reduced (MD = −0.36, 95% CI (−0.60, −0.11), *P*=0.005), as shown in Figure 10. In order to clarify the sources of heterogeneity, the sensitivity analysis was used to exclude the literature one by one. When Feng (2018) was excluded (*P*=0.38, *I*^2^ = 0%), this study is considered the source of heterogeneity. From the original text, it is found that the intervention course of this study is shorter than that of the other two studies. A total of four studies reported LDL-C [16, 18, 26], and three studies reported HDL-C [16–18]. The significant heterogeneity was found after tests (*P* < 0.00001, *I*^2^ = 98%; *P*=0.009, *I*^2^ = 79%). The pooled results showed that compared with the control group, the experimental group of SBP could significantly reduce LDL-C (MD = −0.67, 95% CI (−1.21, −0.12), *P*=0.02) and increase HDL-C (MD = 0.18, 95% CI (0.03, 0.33), *P*=0.02), through sensitivity analysis and subgroup analysis, there was no significant change in the heterogeneity of LDL-C and HDL-C, which was speculated to be related to the differences in drug types, dosages, and administration times, as shown in Figure 11 and Figure 12.

#### 3.4.6. Adverse Reactions

**Figure 13:**
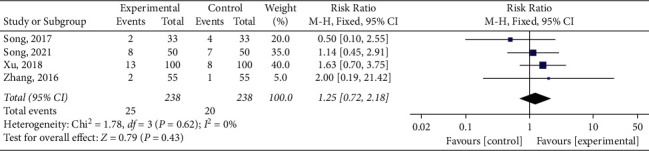
Meta-analysis of adverse reactions.

Four eligible studies reported the incidence of adverse reactions [16, 23, 28, 29], including gastrointestinal intolerance, tongue numbness, dizziness, and rash (Table 2). Heterogeneity was not found in the studies (*P*=0.62, *I*^2^ = 0%). A fixed-effect model performed a meta-analysis. The pooled results showed no significant statistical significance in the incidence of adverse reactions between the two groups (RR = 1.25, 95% CI (0.72, 2.18), *P*=0.43), as shown in Figure 13.

#### 3.4.7. Evaluation of Publication Bias

**Figure 14:**
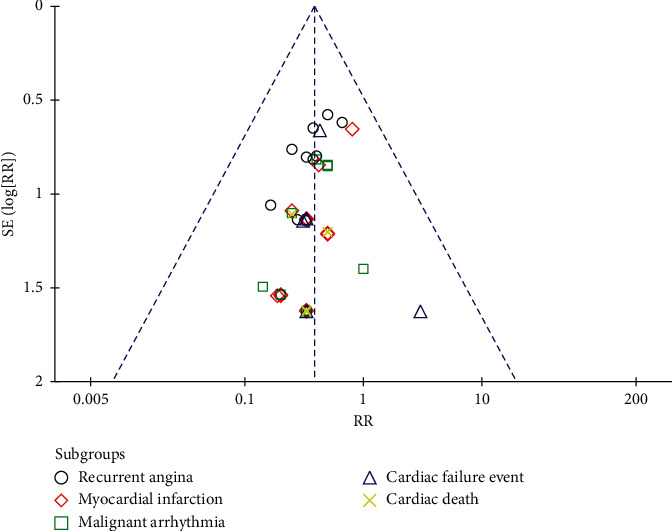
Funnel plot of major adverse cardiac events.

The funnel plot can be used to assess whether there is publication bias in the observed data, and the publication bias of MACE is evaluated. As shown in Figure 14, the left and proper distribution of scattered points are relatively symmetrical, indicating a slight bias of the included research publication.

## 4. Discussion

With the acceleration of population aging and the change of people's *s* living habits, the prevalence and mortality of cardiovascular diseases in China keep growing. The number of patients has reached 290 million. The death toll accounts for more than 40% of the total number of deaths from diseases of the residents, which is the first among all diseases, endangering the health of the people seriously [33]. With the development of PCI, many CHD patients benefit from it. However, the residual risk of patients with CHD after PCI is still high, so long-term management is essential. In China, Shexiang Baoxin Pill is usually combined with conventional western medicine treatment, which is increasingly used for the long-term treatment of CHD after PCI. The role of this treatment requires further comprehensive and systematic evaluation.

This research aims at evaluating the efficacy and safety of SBP in the treatment of CHD after PCI and observing the indices of MACE, LVEF, NT-pro-BNP, inflammatory mediators, blood lipid index, and adverse reactions. As far as we know, this is the first systematic review and meta-analysis of the long-term management of SBP in CHD after PCI. The pooled data showed that the addition of SBP into routine treatment in patients with CHD after PCI might have some beneficial effects on some indicators. Since the incidence of MACE after PCI is still high (2.1–19%) [34], reducing the risk of MACE should be one of the main objectives of long-term management of CHD after PCI. In this systematic review, it was found that SBP had potential advantages in improving the incidence of MACE (recurrent angina, myocardial infarction, malignant arrhythmia), LVEF, NT-pro-BNP, inflammatory mediators (IL-6, hs-CRP), and blood lipid index (TC, TG, LDL-C, HDL-C) in patients with CHD after PCI. There was no significant difference in the overall incidence of adverse reactions between the two groups. The most common adverse reactions of long-term use of SBP are gastrointestinal intolerance and tongue numbness. There is no withdrawal due to severe side effects in the eligible studies. Before more eligible studies are included, only the relative safety of SBP can be temporarily determined without increasing the incidence of adverse reactions. In addition, the subgroup analysis results showed that the intervention course of the combined therapy might be the source of heterogeneity of NT-pro-BNP and hs-CRP.

This systematic review described the related indicators of CHD after PCI precisely and objectively, and there are still some potential limitations to be considered: first of all, a total of 19 eligible studies declared randomization, but some did not describe specific random methods. Researchers ' allocation concealment in all eligible studies is unclear, leading to selection bias. None of the studies explained the sample size calculation method, and the sample size was not generally significant. The lack of a large sample and multicenter RCTs reduces research results' evidence intensity and recommendation level. Secondly, the intervention treatment of the control group was classified as a routine treatment, and the differences in the types, doses, and frequencies of specific drugs were not clarified, resulting in increased clinical heterogeneity. The inclusion criteria are not uninformed, and there is no clear distinction between acute coronary syndrome, angina pectoris, myocardial infarction, and whether combined with underlying diseases. The duration and severity of the disease may be different, which also increases the clinical heterogeneity. Finally, no matter what treatment methods were adopted, the ultimate goal was to improve the long-term prognosis of patients. Most of the eligible studies were not followed up, which could not reflect the long-term efficacy of the combined medication and its impact on patients' long-term quality of life. The design and implementation of high-quality clinical research is the key to improving the intensity of evidence. In future studies, the design, implementation, and publication of clinical trials should be strictly regulated, such as reasonable sample size estimation and registration of clinical research programs before trials, precise random methods, implementation of allocation concealment and blinding, use of placebo controls, detailed records of shedding cases, and use of intention-to-treat analysis reports. In addition, we encourage the publication of negative results.

## 5. Conclusion

The available evidence suggests that SBP could demonstrate a beneficial role in patients with CHD after the PCI of reducing the incidence of MACE and improving LVEF, NT-pro-BNP, inflammatory mediators, and blood lipid index. However, due to the overall quality of the eligible studies, more rigorously designed and standardized, high-quality randomized controlled trials are expected to verify the clinical efficacy of SBP in the treatment of CHD after PCI.

## Figures and Tables

**Table 1:** Basic characteristics of literature research.

Study ID	Sample size		Age (year)		Duration	Follow-up time	Intervention	Control	SBP dosage	Outcomes
	T	C	T	C						
Sun, [14]	64	64	72.55 ± 3.63	72.34 ± 3.46	6 months	NR	SBP + control	PCI + CT	2 pills, t.i.d.	①
Huang, [15]	30	30	56.81 ± 12.61	55.11 ± 12.32	12 months	NR	SBP + control	PCI + CT	2 pills, t.i.d.	④
Xu, [16]	100	100	59.76 ± 10.43	59.36 ± 10.13	3 months	12 months	SBP + control	PCI + CT	1 pill, t.i.d.	①④⑤⑥
Feng, [17]	45	45	62.8 ± 5.1	62.5 ± 4.8	1 month	12 months	SBP + control	PCI + CT	3 pills, t.i.d.	①④⑤
Si, [18]	60	60	50.13 ± 11.27 (total)		6 months	18 months	SBP + control	PCI + CT	2 pills, t.i.d.	①④⑤
Jia, [19]	37	35	55.00 ± 10.73	60.31 ± 11.67	6 months	6 months	SBP + control	PCI + CT	2 pills, t.i.d.	③
Hu, [20]	50	50	58.0 ± 8.7(total)		12 months	NR	SBP + control	PCI + CT	2 pills, t.i.d.	①
Gao, [21]	51	51	NR		12 months	12 months	SBP + control	PCI + CT	2 pills, t.i.d.	①②
Zhang, [22]	60	50	48.10 ± 5.12	48.15 ± 5.10	6 months	NR	SBP + control	PCI + CT	2 pills, b.i.d.	①④
Song, [23]	50	50	56.4 ± 8.6	55.9 ± 7.7	3 months	NR	SBP + control	PCI + CT	2 pills, t.i.d.	①②③⑥
Yu, [24]	60	60	62.41 ± 4.33	62.53 ± 4.42	1 month	NR	SBP + control	PCI + CT	2 pills, t.i.d.	②
Wei, [25]	36	36	65.25 ± 6.30	64.90 ± 6.16	3 months	NR	SBP + control	PCI + CT	2 pills, t.i.d.	②④
Ran, [26]	100	100	61.00 ± 10.33	62.31 ± 12.07	12 months	NR	SBP + control	PCI + CT	2 pills, t.i.d.	③⑤
Jia, [27]	38	38	61.3 ± 8.9	59.8 ± 9.1	2 months	NR	SBP + control	PCI + CT	3 pills, t.i.d.	①
Zhang, [28]	55	55	60.5 ± 9.1	61.7 ± 10.3	4 weeks	NR	SBP + control	PCI + CT	2 pills, t.i.d.	①②③⑥
Song, [29]	33	33	73.92 ± 8.11	73.29 ± 8.03	16 weeks	NR	SBP + control	PCI + CT	1 pill, t.i.d.	①②⑥
Shi, [30]	40	40	66.8 ± 6.9	68.2 ± 7.5	3 months	NR	SBP + control	PCI + CT	2 pills, t.i.d.	①②
Ma, [31]	60	56	62.17 ± 8.33	61.96 ± 7.45	4 weeks	3 months	SBP + control	PCI + CT	2 pills, t.i.d.	①②
Sun [32]	50	50	61 ± 3	61 ± 3	1 week	4 weeks	SBP + control	PCI + CT	2 pills, t.i.d.	①②

Notes: *T*, trial group; C, control group; NR, not report; SBP, Shexiang Baoxin Pill; PCI, percutaneous coronary intervention; CT, conventional treatment; t.i.d., three times a day; b.i.d., two times a day; ① MACE ② LVEF; ③ NT-pro-BNP; ④ inflammatory mediators; ⑤ blood lipid index; ⑥ adverse reactions.

**Table 2:** Adverse reactions.

Studies	Adverse drug reactions or adverse events	
	T	C
Xu, [16]	4 cases of gastrointestinal intolerance, 2 cases of dizziness, 3 cases of abnormal liver and kidney function, 4 cases of tongue numbness	3 cases of gastrointestinal intolerance, 3 cases of dizziness, 2 cases of abnormal liver and kidney function
Song, [23]	2 cases of gastrointestinal intolerance, 1 case of rash, 3 cases of bleeding, 2 cases of tongue numbness	2 cases of gastrointestinal intolerance, 1 case of rash, 3 cases of bleeding, 2 cases of tongue numbness
Zhang, [28]	1 case of gastrointestinal intolerance, 1 case of rash	1 case of gastrointestinal intolerance
Song, [29]	1 case of gastrointestinal intolerance, 1 case of fever	2 cases of gastrointestinal intolerance, 1 case of fever, 1 case of rash

## Data Availability

According to reasonable requirements, the datasets used and analyzed in the current study can be obtained from the corresponding authors.
